# Supplementary material for: Increased glucose availability sensitizes pancreatic cancer to chemotherapy
Source: Nat Commun. 2023 Jun 28;14:3823. doi: 10.1038/s41467-023-38921-8 (PMC10307839; doi:10.1038/s41467-023-38921-8)
Supplement: Supplementary file 1 — Supplementary Information [file 41467_2023_38921_MOESM1_ESM.pdf]

Supplementary Information

Increased glucose availability sensitizes pancreatic cancer to chemotherapy

Ali Vaziri-Gohar, Jonathan J. Hue, Ata Abbas, Hallie J. Graor, Omid Hajihassani, Mehrdad Zarei, George Titomihelakis, John Feczko, Moeez Rathore, Sylwia Chelstowska, Alexander W. Loftus, Rui Wang, Mahsa Zarei, Maryam Goudarzi, Renliang Zhang, Belinda Willard, Li Zhang, Adam Kresak, Joseph E. Willis, Gi-Ming Wang, Curtis Tatsuoka, Joseph M. Salvino, Ilya Bederman, Henri Brunengraber, Costas A. Lyssiotis, Jonathan R. Brody, Jordan M. Winter

Supplementary Figures and Legends 1 to 7

Supplementary Tables 1 and 2

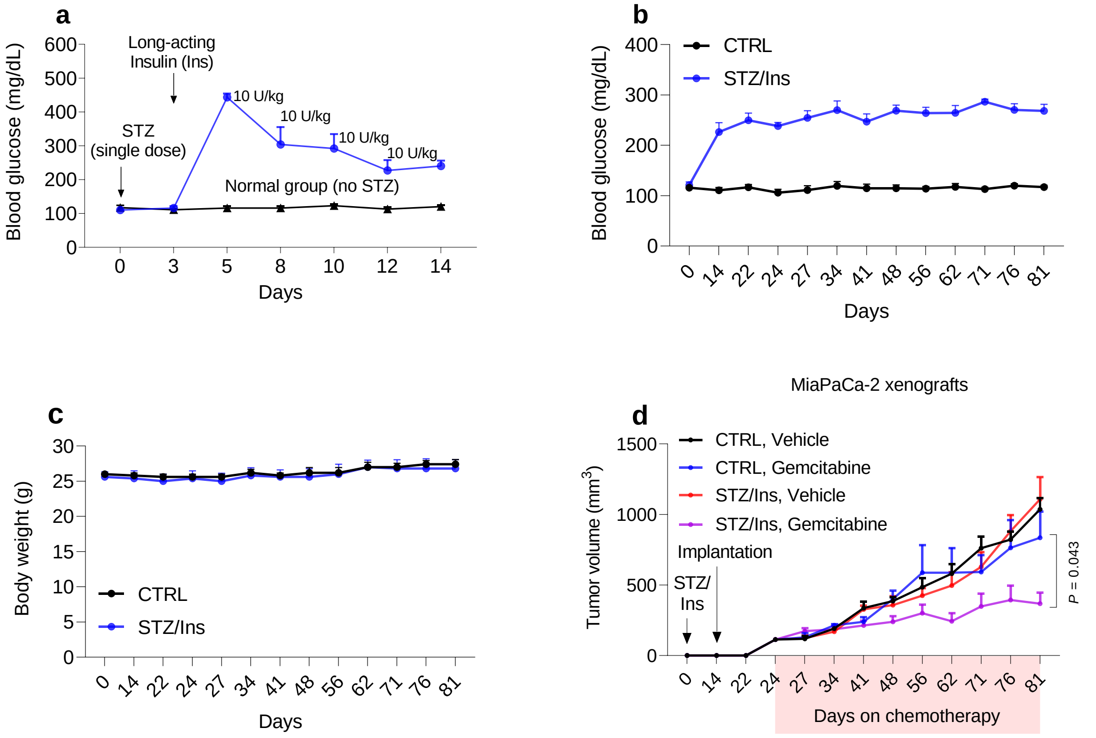

Supplementary Figure 1 | Hyperglycemic induced by streptozotocin results in superior response to conventional chemotherapy. a. Pharmacologically induced hyperglycemia in nude mice with a single dose of streptozotocin (STZ, 120 mg/kg) followed by daily subcutaneous insulin glargine injections to keep glucose levels in a non-toxic, but still hyperglycemic range (n=5 mice per group). **b, c,** Peripheral glucose levels (**b**) and body weights (**c**) of nude mice that received normal water or STZ with insulin glargine (STZ/Ins). Each dot represents the mean of weekly measurements of blood glucose per group (n=5 mice per group). **d**, Xenograft growth of MiaPaCa-2 cells treated with gemcitabine (n=5 mice per group)**.** Data are provided as mean ± s.e.m. Longitudinal mixed models were fit for tumor size growth, and time by treatment interactions were assessed (**d**). Source data are provided as a Source Data file.

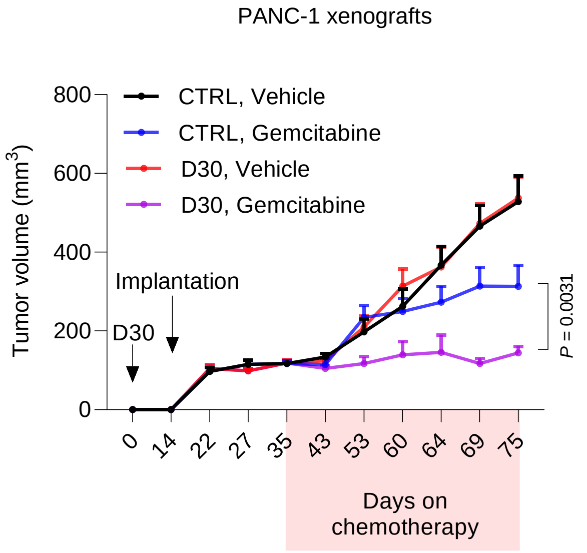

Supplementary Figure 2 | PANC-1 xenografts respond better to gemcitabine under high glucose diet. Xenograft growth of PANC-1 cells treated with gemcitabine (n=5 mice per group, 75 mg/kg twice weekly, i.p.)**.** Data are provided as mean ± s.e.m. Longitudinal mixed models were fit for tumor size growth, and time by treatment interactions were assessed.

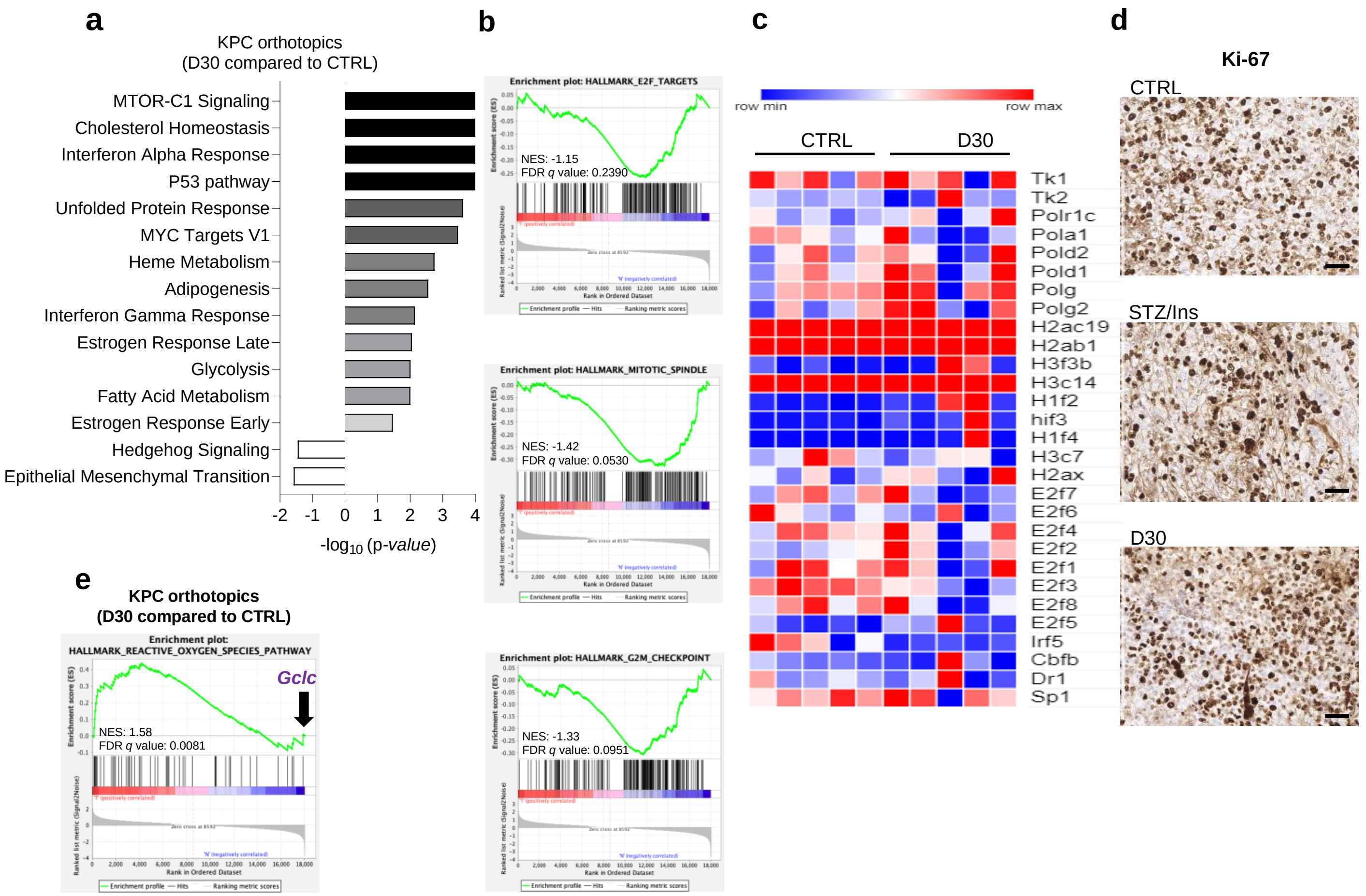

Supplementary Figure 3 | Altered biology and biochemistry in pancreatic orthotopic tumors from hyperglycemic mice. a, Enriched cellular processes, derived from transcriptomic analyses, in KPC orthotopic tumors (D30 compared to Ctrl) (n=5 orthotopic tumors per group). **b, c,** GSEA (**b**) and heatmap (**c**) of genes associated with DNA replication and cell cycle division in KPC orthotopic tumors under the indicated conditions (n=5 orthotopic tumors). **d**, Ki-67 immunoblotting of MiaPaCa-2 xenograft tumors under indicated conditions for 14 days (representative immunoblots of three tumors with similar results are shown). FDR-adjusted p-value (q values) are provided. Scale bars, 50 µm. **e**, GSEA of genes associated with oxidative stress in KPC orthotopic tumors under D30 compared to Ctrl (n=5 orthotopic tumors per group).

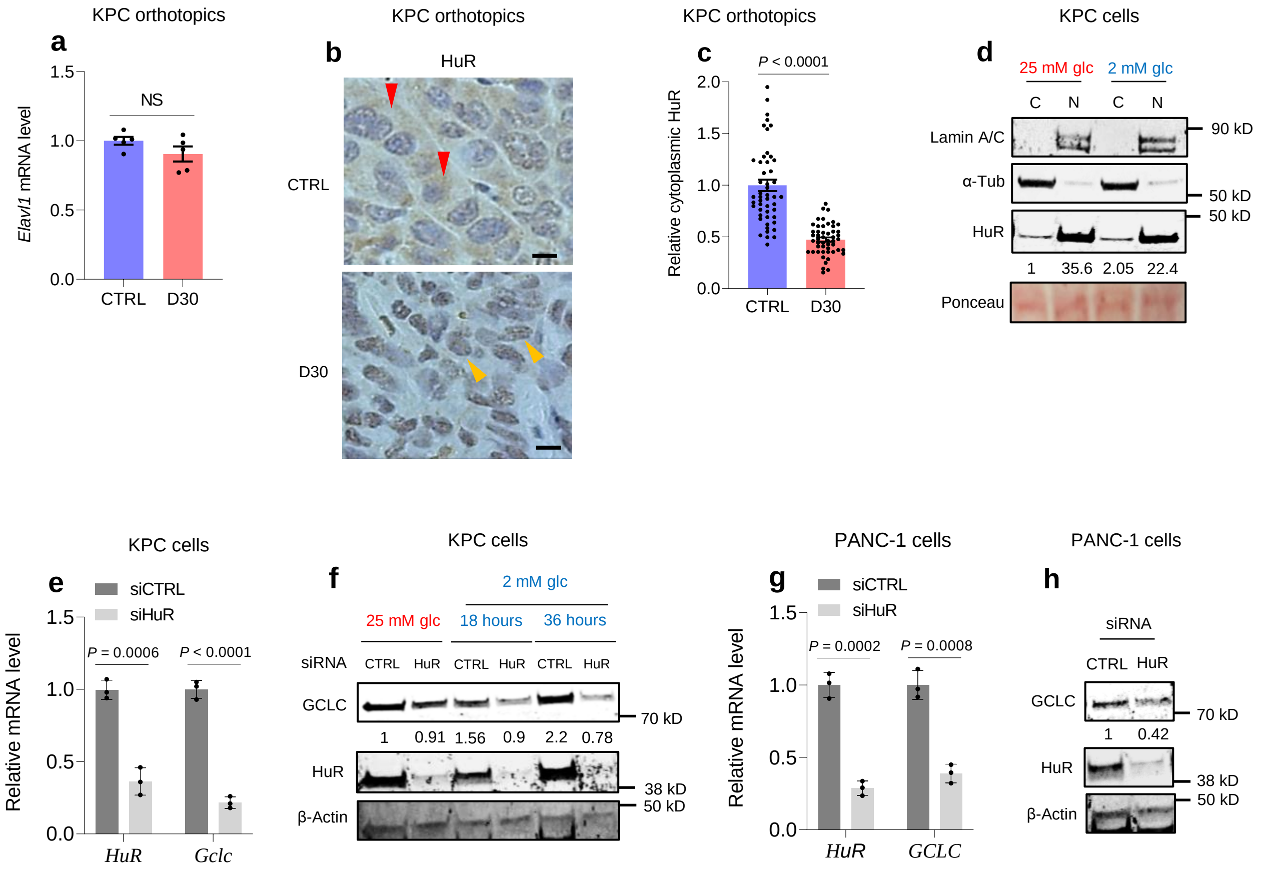

Supplementary Figure 4 | HuR regulates *GCLC* expression in pancreatic cancer. a, qPCR analysis of *Elavl1* (HuR) in KPC orthotopic tumors under the indicated conditions (n=5 tumors per group). **b, c**, Immunohistochemistry immunolabeling of HuR (**b,** representative immunoblots of five tumors with similar results are shown) and analysis of subcellular localization of HuR (n=50 cells per group) (**c**) in KPC orthotopic tumors from mice receiving D30 water or normal water. Hematoxylin staining was used to label nuclei. Scale bars, 10 µm. **d**, Representative immunoblots of three independent experiments with similar results in KPC cells under the indicated conditions for 48 hours. **e-h,** qPCR analysis (**e, g,** n=3 independent experiments) and representative immunoblots of two independent experiments with similar results after siRNA silencing of HuR in KPC cells under indicated conditions (**f**) and in PANC-1 under low glucose conditions for 36 hours (**h**). Data are provided as mean ± s.d. (**e, j**) or mean ± s.e.m. (**a**, **c**). Pairwise comparisons were conducted using two-tailed, unpaired Student's *t*-tests. Source data are provided as a Source Data file.

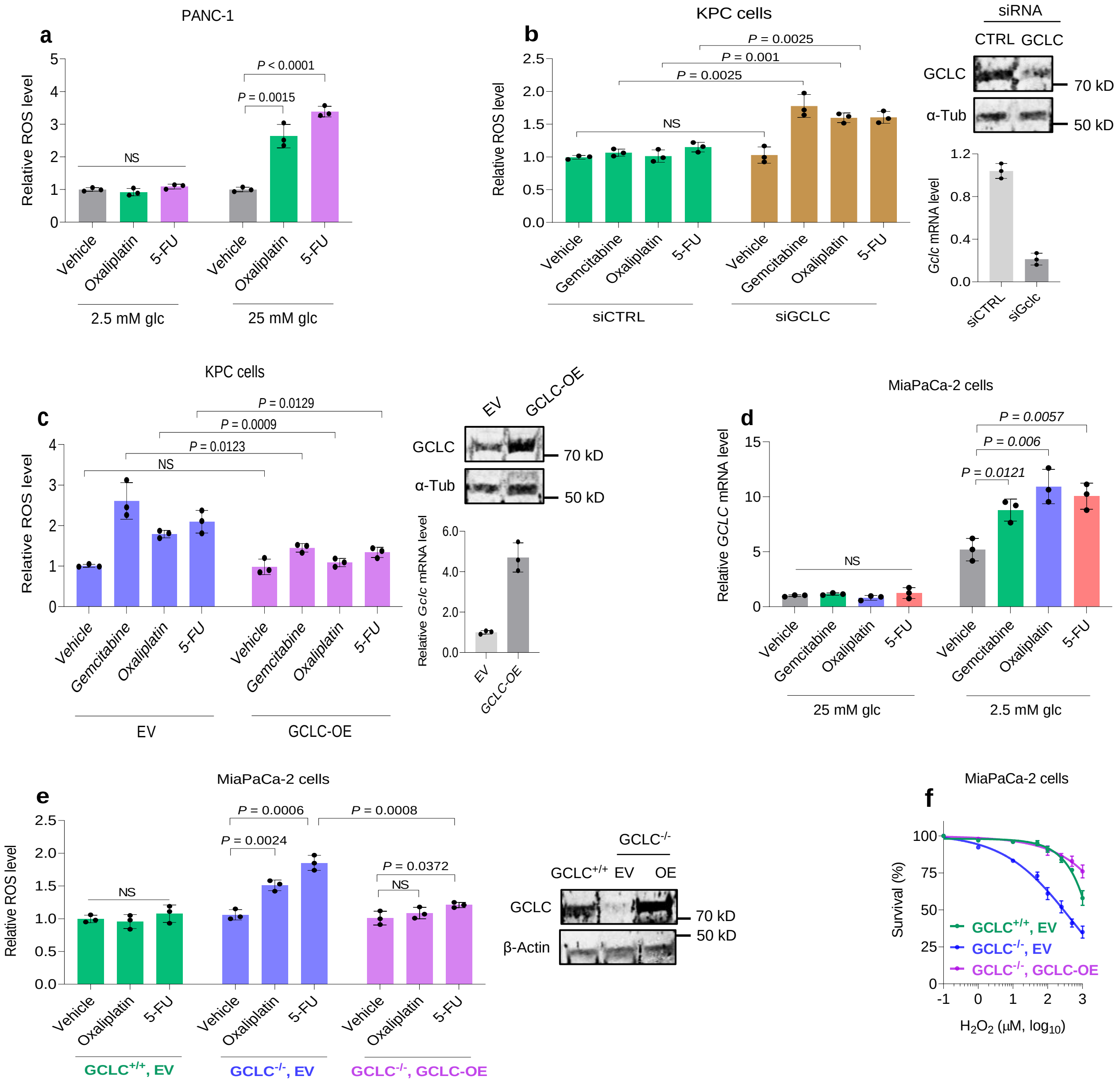

Supplementary Figure 5 | a-c, ROS levels in PANC-1 cells under indicated conditions for 30 hours followed by administration of indicated chemotherapies (1 μM) for an additional 16-18 hours (**a**), transiently transfected with siRNAs (control non-targeting or against *GCLC*) cultured in low glucose (2 mM) conditions for 30 hours (**b**) or transiently transfected with *GCLC* overexpressing plasmid and cultured in high glucose (25 mM) (**c**) followed by chemotherapy administration (gemcitabine (100 nM), oxaliplatin (1 μM), 5-FU (1 μM)) for an additional 16-18 hours. Relative *Gclc* mRNA levels (n= 3 independent experiments) and immunoblots of GCLC (representative of two independent experiments with similar results) are provided. **d**, qPCR analysis of *GCLC* transcripts in MiaPaCa-2 cells under indicated conditions for 48 hours (gemcitabine (10 nM), oxaliplatin (1 μM), 5-FU (1 μM), n= 3 independent experiments). **e**, **f**, Immunoblot of GCLC (representative of two independent experiments with similar results) and relative ROS levels (**e**) and survival of $GCLC^{+/+}$ and $GCLC^{-/-}$ (knockout clone 2) MiaPaCa-2 cells (**f**), after transient transfection with empty vector or GCLC-overexpressing plasmid, under low glucose conditions (2.5 mM) followed by chemotherapy administration (oxaliplatin (1 μM), 5-FU (1 μM)) for an additional 16-18 hours (**e**), or H_2O_2 under low serum conditions (2%) for four days (**f**) (n= 3 independent experiments). Data are provided as mean ± s.d. Pairwise comparisons were conducted using two-tailed, unpaired Student's *t*-tests. Source data are provided as a Source Data file.

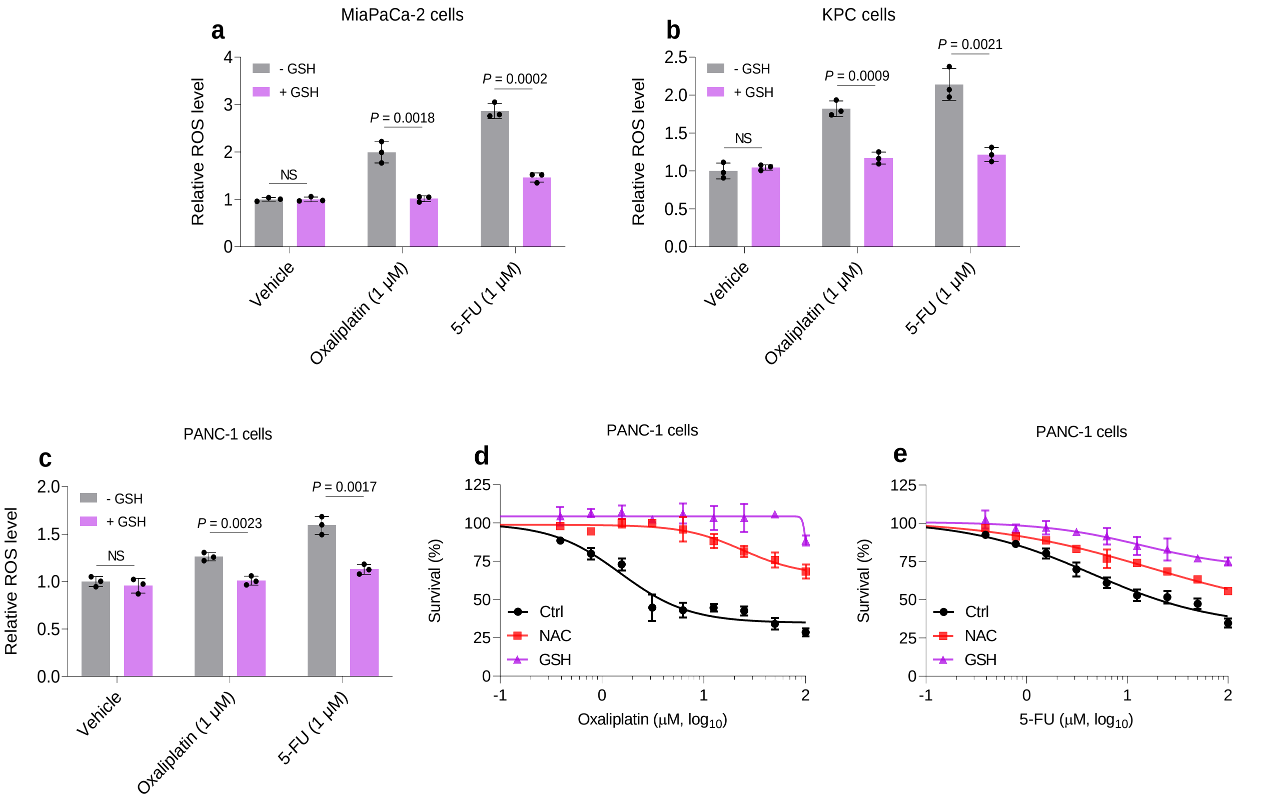

Supplementary Figure 6 | a-c, Relative ROS levels in MiaPaCa-2 (**a**), KPC (**b**), and PANC-1 (**c**) cells cultured in high (25 mM) glucose with or without GSH (4 mM) for 16 hours followed by chemotherapy administration at indicated dose for an additional 18 hours (n= 3 independent experiments). **d, e,** Relative survival of PANC-1 cells cultured in 25 mM glucose under indicated chemotherapy treatments for five days (n= 3 independent experiments). Data are provided as mean ± s.d. Pairwise comparisons were conducted using two-tailed, unpaired Student's *t*-tests. Source data are provided as a Source Data file.

a

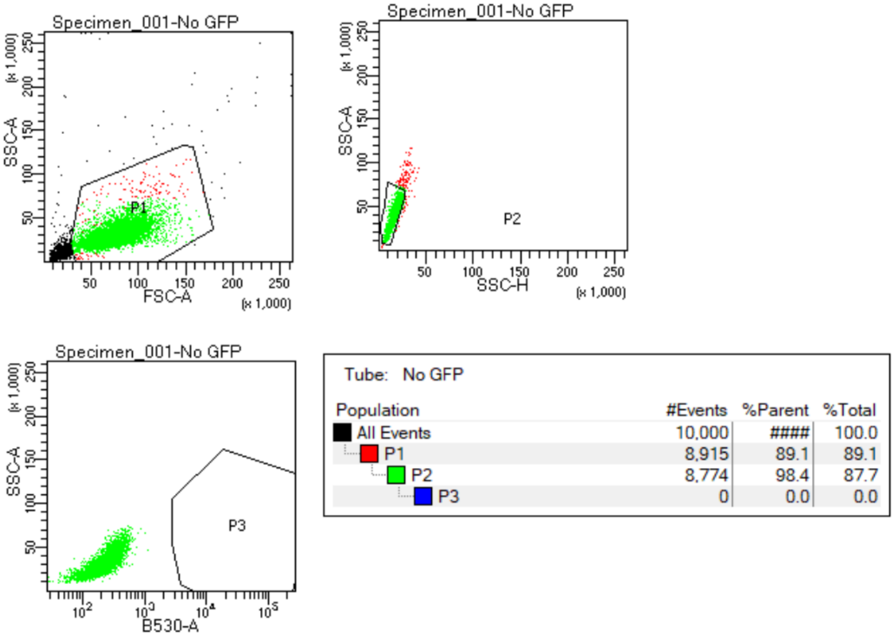

Tube: No GFP

Population	#Events	%Parent	%Total
All Events	10,000	####	100.0
P1	8,915	89.1	89.1
P2	8,774	98.4	87.7
P3	0	0.0	0.0

b

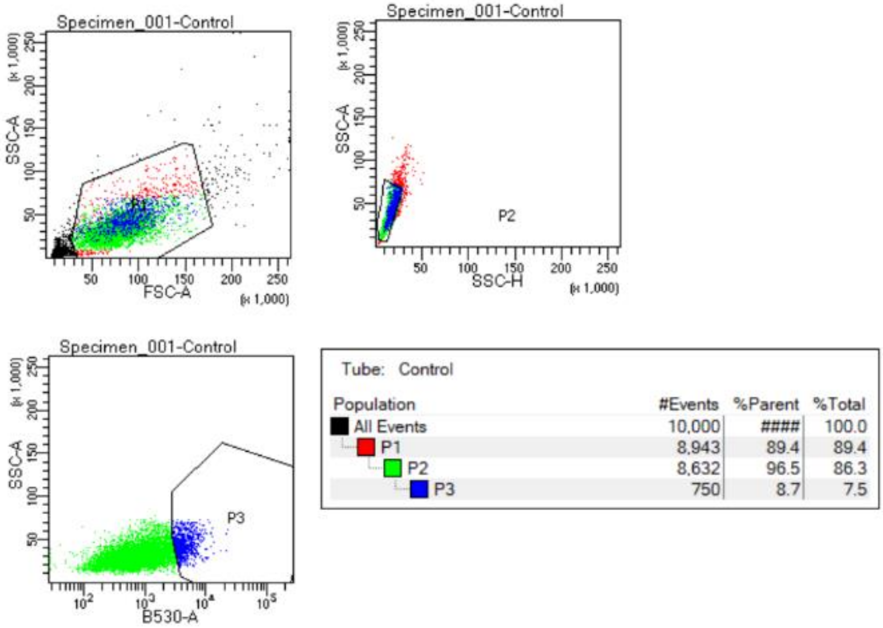

Tube: Control

Population	#Events	%Parent	%Total
All Events	10,000	####	100.0
P1	8,943	89.4	89.4
P2	8,632	96.5	86.3
P3	750	8.7	7.5

Supplementary Figure 7 | FACS gating strategy. **a,** Control sample (no GFP-expressing cells). **b,** Test sample (GFP-expressing cells).

Supplementary Table 1 | Demographic and clinical data of patients with stage IV pancreatic ductal adenocarcinoma, who received chemotherapy or supportive care (no chemotherapy), stratified by normal or high glucose

	Received chemotherapy			Did not receive chemotherapy		
	**Normal glucose**	**High glucose**	***P* value**	**Normal glucose**	**High glucose**	***P* value**
N	135 (56.3%)	105 (43.7%)	---	98 (60.9%)	63 (39.1%)	---
Age, years (median, IQR)	67 (58, 72)	65 (58, 74)	0.94	72 (62, 80)	73 (65, 81)	0.66
Male sex	67 (49.6%)	54 (51.4%)	0.78	48 (49.0%)	31 (49.2%)	0.98
White race	99 (73.3%)	75 (71.4%)	0.90	68 (69.4%)	40 (63.5%)	0.08
ECOG performance status			**0.04**			0.13
≤1	94 (69.6%)	76 (72.4%)		23 (23.5%)	17 (27.0%)	
≥2	13 (9.6%)	18 (17.1%)		21 (21.4%)	21 (33.3%)	
Not recorded	28 (20.7%)	11 (10.5%)		54 (55.1%)	25 (39.7%)	
Charlson comorbidity score (median, IQR)	8 (7, 9)	9 (8, 10)	**0.01**	9 (8, 10)	10 (9, 11)	**0.01**
Documented diabetes at diagnosis	20 (14.8%)	59 (56.2%)	**<0.001**	21 (22.3%)	44 (69.8%)	**<0.001**
Initial metastatic disease site			0.16			0.35
Liver	80 (59.3%)	66 (62.9%)		61 (66.3%)	26 (55.7%)	
Peritoneal cavity	15 (11.1%)	9 (8.6%)		3 (3.3%)	4 (8.2%)	
Multiple sites	30 (22.2%)	15 (14.3%)		19 (20.6%)	11 (27.9%)	
Other, single site	10 (7.4%)	15 (14.3%)		9 (9.8%)	4 (8.2%)	
CA 19-9 at diagnosis, U/mL (median, IQR)	1294.5 (162.4, 7446.2)	2439.5 (246.2, 14318.8)	0.22	1910.8 (95.0, 30099.1)	1715.0 (220.5, 19982.9)	0.70
First-line chemotherapy regimen			0.88			
FOLFIRINOX	51 (37.8%)	35 (33.3%)				
Gemcitabine with nab-paclitaxel	38 (28.2%)	34 (32.4%)				
Gemcitabine	15 (11.1%)	10 (9.5%)				
FOLFOX	20 (14.8%)	15 (14.3%)				
Other	11 (8.1%)	11 (10.5%)				
Number of cycles, first-line (median, IQR)	3 (1, 6)	4 (2, 8)	0.24	0	0	---
Total number of cycles (median, IQR)	5 (2, 10)	7 (2, 13)	0.05	0	0	---

Abbreviations: IQR, interquartile range; ECOG, Eastern Cooperative Oncology Group; FOLFIRINOX (folinic acid, 5-FU, oxaliplatin, irinotecan); nab, nanoparticle albumin-bound; FOLFOX (folinic acid, 5-FU, oxaliplatin). Bold values indicate statistical significance.

Supplementary Table 2 | Multivariable Cox proportional hazards regression analyzing factors associated with overall survival. Separate regressions were performed for patients who received chemotherapy and those who did not receive chemotherapy

	**Received chemotherapy**				**Did not receive chemotherapy**			
	**HR**	**95% CI**		***P* value**	**HR**	**95% CI**		***P* value**
High glucose vs normal glucose	0.61	0.41	0.92	**0.02**	0.99	0.64	1.53	0.97
Modified Charlson comorbidity index*: ≥10 vs ≤9	0.70	0.42	1.16	0.17	0.92	0.63	1.34	0.66
Documented diabetes at diagnosis	1.41	0.93	2.15	0.11	1.00	0.64	1.57	0.99
ECOG performance status								
≤1	Ref.	---	---	---	Ref.	---	---	---
≥2	0.72	0.41	1.26	0.25	1.25	0.75	2.09	0.40
Unknown	1.29	0.80	2.08	0.30	1.34	0.87	2.06	0.19
Initial metastatic disease site								
Liver	Ref.	---	---	---	Ref.	---	---	---
Peritoneum	0.79	0.46	1.38	0.42	1.10	0.51	2.38	0.81
Multiple sites	1.26	0.82	1.93	0.28	1.27	0.85	1.94	0.28
Other, single site	0.48	0.28	0.85	**0.01**	0.62	0.32	1.18	0.15
CA 19-9 at diagnosis								
<37 U/mL	0.98	0.64	1.51	0.94	0.92	0.56	1.51	0.73
37 - 1000 U/mL	0.76	0.50	1.14	0.18	0.59	0.35	0.98	**0.04**
>1000 U/mL	Ref.	---	---	---	Ref.	---	---	---
Unknown	0.52	0.28	0.98	**0.04**	0.98	0.62	1.53	0.91
Total number of cycles: ≥10 vs ≤9**	0.15	0.10	0.22	**<0.001**				
First-line chemotherapy	0.84	0.59	1.18	0.32				

*Modified Charlson comorbidity index does not include diabetes mellitus, as this was analyzed separately in multivariable models.
**Patients who received ≤1 cycle of chemotherapy were excluded from analyses.
Abbreviations: IQR, interquartile range; ECOG, Eastern Cooperative Oncology Group; FOLFIRINOX (folinic acid, 5-FU, Oxaliplatin, irinotecan); nab, nanoparticle albumin-bound; HR, hazard ratio; CI, confidence interval; Ref, reference.
First-line chemotherapy: FOLFIRINOX or gemcitabine with nab-paclitaxel vs other.
Bold values indicate statistical significance.
